# Supplementary figures and images for: Effect of Cryopreservation and Post-Cryopreservation Somatic Embryogenesis on the Epigenetic Fidelity of Cocoa (Theobroma cacao L.)
Source: PLoS One. 2016 Jul 12;11(7):e0158857. doi: 10.1371/journal.pone.0158857 (PMC4942035; doi:10.1371/journal.pone.0158857)

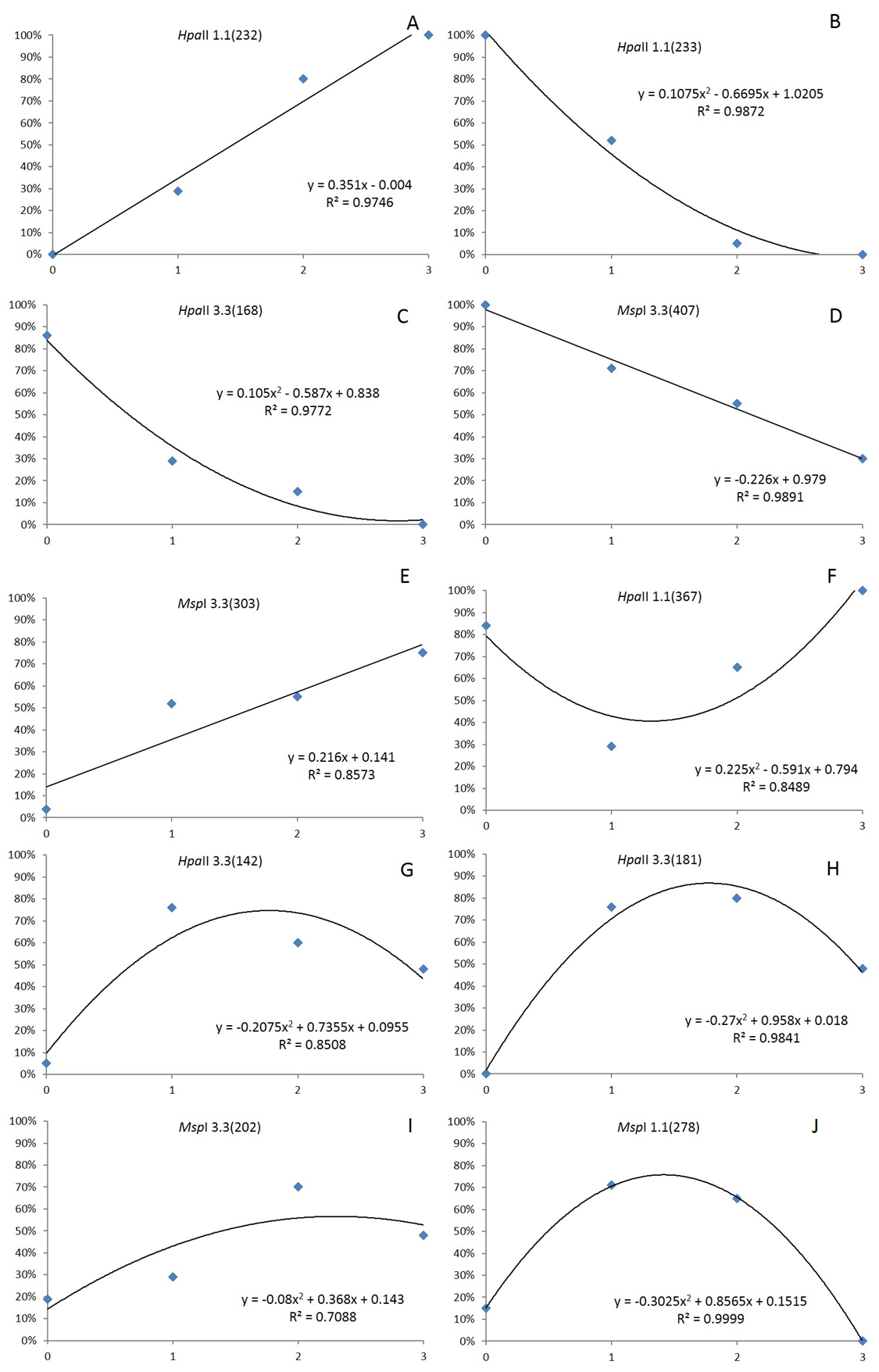

Supplement: S1 Fig — Values on the horizontal axis indicated the number of treatments each group has been subjected to (0 = Donor Plant, 1 = secondary somatic embryos maintained in ED medium, 2 = secondary somatic embryos after cryopreservation for 1 h in liquid nitrogen (1h LN) and 3 = tertiary somatic embryos generated from 1 h LN samples). Epilocus frequencies were calculated from presence/absence MSAP profiles generated combining Hp3/Eco3 and Hp1/Eco1 selective primer combinations and restriction enzymes HapII and MspI using GenAlex 6.1 software. (TIF) [file pone.0158857.s001.tif]
